# Data on microcirculatory perfusion dips in the resting nail bed

**DOI:** 10.1016/j.dib.2018.10.077

**Published:** 2018-10-27

**Authors:** Robin Mirdell, Aukje Nienke Lemstra-Idsardi, Simon Farnebo, Erik Tesselaar

**Affiliations:** a Department of Clinical and Experimental Medicine, Faculty of Health Sciences, Linköping University, Linköping, Sweden; b Department of Radiation Physics, Department of Medical and Health Sciences, Linköping University, Linköping, Sweden; c Department of Plastic Surgery, Hand Surgery, and Burns, Linköping University, Linköping, Sweden; d University of Twente, Enschede, Netherlands

**Keywords:** laser speckle contrast imaging, nail bed, microcirculation, perfusion

## Abstract

This article contains the raw data from the article entitled: “The presence of synchronized perfusion dips in the microcirculation of the resting nail bed” Mirdell et al. (in press). A laser speckle contrast imager (LSCI) was used to make a total of 21 recordings of the perfusion in the resting nail bed of 10 healthy test subjects. The first 10 recordings were acquired after 5 min of acclimatization. An additional 10 recordings were acquired in the same test subjects, after 20 min of acclimatization. In the last recording, a digital nerve block was applied to the left dig III. The data show the presence of highly irregular perfusion variations, a phenomenon we like to call perfusion dips. The data also show how the perfusion dips can be abolished through a digital nerve block. An algorithm for the quantification of the perfusion dips is included in the data.

**Specifications table**

**Table:** 

Subject area	*Biology*
More specific subject area	*Microcirculation*
Type of data	*Perfusion data as raw signals and images.*
How data was acquired	*Data were acquired using a laser speckle contrast imager (PeriCam PSI System, Perimed AB, Järfälla, Sweden)*
Data format	*Data are provided as MS Excel files and in JPEG format. Both raw perfusion and calculations from the perfusion dip algorithm is included in the material.*
Experimental factors	*Noninvasive perfusion recording. Digital nerve block and subsequent perfusion recording. Different seated duration in the same test subjects.*
Experimental features	*The perfusion in the resting nail bed was recorded in 10 healthy test subjects in two different sessions. In the 1*^*st*^*session the test subject had been seated for 5 min and in the 2*^*nd*^*session for 20 min. A digital nerve block was applied in a single test subject to investigate mechanism behind observed irregular perfusion dips.*
Data source location	*Linköping University, Sweden.*
Data accessibility	*The data are available in this article.*

**Value of the data**

• This shows the natural occurrence of perfusion dips in the resting nail bed of healthy test subjects at a high temporal resolution over a 20 min duration.
• The raw data were analyzed with a peak detection algorithm to successfully identify the irregular perfusion dips and to quantify them. Both the algorithm and the raw data from all recordings are available.
• Two image series, which clearly show perfusion dips, are also included in the material.
• The data allow other researchers to improve upon the rudimentary perfusion algorithm presented herein.

## 1 Data

**Fig. 1:**
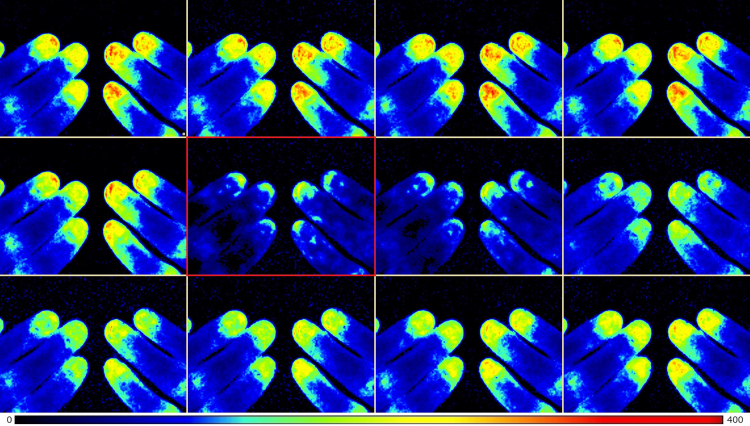
Perfusion image series captured 15 min after the start of the 2^nd^ session in the most representative test subject (#10). Each image is captured 4 s apart. The series start on the upper left and ends on the lower right. The first perfusion images show both hands in a vasodilatory state. The perfusion image encircled by red, marks the perfusion nadir.

**Fig. 2:**
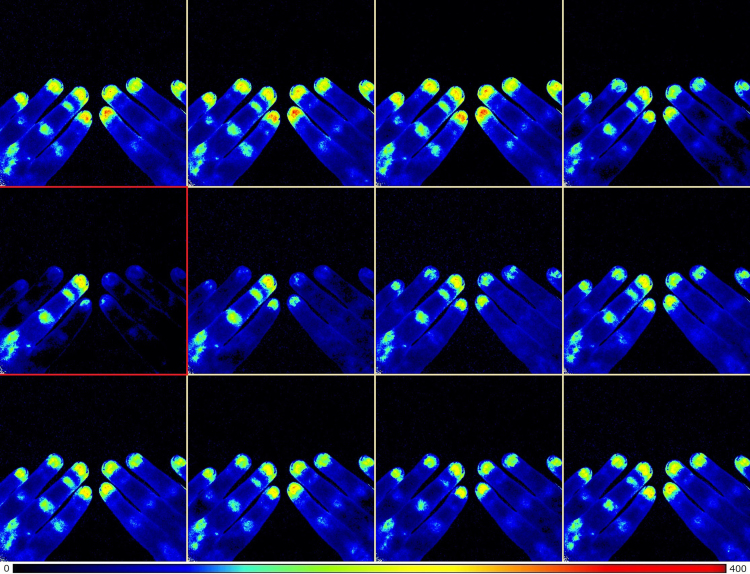
Perfusion image series captured 17 min after the application of digital nerve block on dig III sin (subject #9). Each image is captured 4 s apart. The image series start on the upper left and ends on the lower right. The first images show both hands in a vasodilatory state. Later images show a clear decrease in perfusion. The image encircled by red, indicates the perfusion nadir. Note how dig III sin remains at an unchanged perfusion level throughout the entire image series.

The data consists of perfusion signals from 21 recordings. Temperature of the digits at the start and end of each session is also included. Perfusion images were recorded using a laser speckle contrast imager (LSCI). Regions of interest (ROI) were then created over the nail bed of digits II-IV in each recording. The perfusion data consist of raw data from each ROI, test subject and recording. Each recording contains roughly 60,000–80,000 perfusion values and the results from the peak detection algorithm is found alongside the raw perfusion signal. Fig. 1, Fig. 2 show an image sequence of raw perfusion images as a perfusion dip occurs, with and without a digital nerve block.

## 2 Experimental design, materials and methods

The LSCI measurements were done to investigate the normal perfusion variations in the microcirculation of the nail bed. Ten healthy test subjects were recruited and none of the participants had any medical conditions to report. The study was approved by the regional ethics review board, DNr 2012/31/31.

A laser speckle contrast imager (PeriCam PSI System, Perimed AB, Järfälla, Sweden) was used to measure the perfusion in the nail bed. The perfusion was recorded at 44 images per second averaged over 4 images resulting in a frequency of 11 Hz. The measurement distance was set to 20–25 cm and at the given distance, the resolution was around 0.6 mm/speckle pixel. The recorded image is color coded where red indicates high perfusion and blue low perfusion [2], [3], [4].

All measurements were done in the same room and the room temperature was kept at 22±1 °C. The experimental procedure was divided into two different sessions. Both sessions were done on different days at least 24 h apart. During the 1st session, each test subject was seated for 5 min before the start of the perfusion measurement. For the 2nd session, the test subjects had been seated for 20 min instead. There was no additional acclimatization period before the seated duration. The perfusion was then measured for 20 min in the nail bed of digits II-IV bilaterally using LSCI. The temperature of the digits was also measured at the start and end of each perfusion measurement.

A dip detection algorithm was designed considering several aspects of the known variation in the microcirculation. The dip detection algorithm is presented alongside the raw perfusion data. For a discussion about the different parts of the formulas, please see the original article [1].

To explore the mechanism behind the perfusion dips, a distal digital nerve block was applied to the third digit of the left hand (dig III sin) in one healthy test subject. Mepivacaine (Carbocain, Aspen Nordic, Ballerup, Denmark) at a concentration of 2% was used and roughly 0.75 ml was injected at each digital nerve. Perfusion was recorded as previously described for 10 min before and 20 min after applying the local anesthesia.
